# Evidence for Potentiation of M-Type Potassium Current by Flavonoid Corylin (3-(2,2-Dimethylchromen-6-yl)-7-hydroxychromen-4-one)

**DOI:** 10.3390/ph19050713

**Published:** 2026-04-30

**Authors:** Sheng-Nan Wu, Rasa Liutkevičienė, Sheng-Che Lin

**Affiliations:** 1 Department of Medical Research, An Nan Hospital, China Medical University, No. 66, Section 2, Changhe Road, An Nan District, Tainan 70965, Taiwan; 2 Laboratory of Ophthalmology, Institute of Neuroscience, Lithuanian University of Health Sciences, Eivenių St. 4, LT-50161 Kaunas, Lithuania; rasa.liutkeviciene@lsmu.lt; 3 Department of Plastic Surgery, An Nan Hospital, China Medical University, Tainan 70965, Taiwan

**Keywords:** corylin (3-(2,2-dimethylchromen-6-yl)-7-hydroxychromen-4-one), M-type K^+^ current, M-type K^+^ (KCNQ/K_V_7) channel, current kinetics, voltage-dependent hysteresis, docking analysis

## Abstract

**Background:** Corylin (3-(2,2-dimethylchromen-6-yl)-7-hydroxychromen-4-one), a bioactive flavonoid, has been reported to exercise anti-inflammatory, antineoplastic, and antioxidant effects, and may also possess lifespan-extending properties. **Objectives:** Any modifications of transmembrane ionic currents produced by corylin remain largely unknown. **Methods:** The patch-clamp technique and docking prediction were used in this study. **Results:** In pituitary GH_3_ somatolactotrophs, corylin concentration-dependently increased the magnitude of the M-type K^+^ current (*I*_K(M)_), with an EC_50_ of 3.8 μM. Concurrently, the activation time constant of *I*_K(M)_ was shortened. The addition of linopirdine (10 μM), an *I*_K(M)_ inhibitor, suppressed the current amplitude. Corylin also induced a leftward shift in the steady-state activation curve and enhanced *I*_K(M)_ during pulse-train stimulation. Moreover, corylin increases the hysteretic strength of *I*_K(M)_ evoked by a long-lasting triangular ramp pulse; this effect was attenuated by linopirdine. The stimulatory effect of corylin on *I*_K(M)_ was not altered by carvedilol or iberiotoxin but was reduced by dapagliflozin. In contrast, depolarization-activated *I*_K(M)_ was not affected by 17β-estradiol alone. In cell-attached recordings, corylin increased M-type K^+^ (K_M_)-channel activity with minimal change in single-channel amplitude, while prolonging the mean open time. This stimulatory effect was reversed by linopirdine or dapagliflozin. Additionally, corylin slightly inhibited the *erg*-mediated current. Docking analysis further suggested that corylin potentially interacts with residues in KCNQ2 or KCNH2 channels via hydrogen bonding and hydrophobic interactions. **Conclusions:** These findings suggest that corylin modulates ionic currents, primarily through K_M_ (KCNQ/K_V_7) channels, which may underlie its in vivo actions and those of related flavonoids. These effects may contribute to the regulation of functional activities of neuronal, neuroendocrine, and endocrine cells.

## 1. Introduction

Corylin (3-(2,2-dimethylchromen-6-yl)-7-hydroxychromen-4-one) is a major bioactive flavonoid isolated from the fruit of *Psoralea corylifolia* Linne (family Fabaceae), also known as buguchi or Bo-Gol-Zhee. It has been increasingly demonstrated to exert a wide range of pharmacological actions, including direct free radical scavenging, inhibition of biomolecules, suppression of lipopolysaccharide-induced inflammation, and modulation of antioxidant defense [1,2,3,4,5,6,7,8,9,10,11,12,13,14,15,16,17,18,19,20]. This compound might also have potential in treating brain inflammation and attenuating the progression of neurodegeneration. For example, the extract from the seeds of *Psoralea corylifolia* has been demonstrated to be effective against palmitate-induced neuronal apoptosis in PC12 cells [21] and to exert neuroprotective and anti-neuroinflammatory effects in hippocampal cells, microglia and retinal ganglion cells [4,22]. Notably, in addition to stimulating L-type Ca^2+^ currents [23,24], quercetin—another flavonoid—has been reported to enhance the activity of M-type K^+^ channels [25,26,27]. Several botanical medicines have also been recently shown to activate KCNQ channels [26,27]. However, the extent to which corylin or other related compounds can alter the magnitude, gating properties, and voltage-dependent hysteresis (Hys_(V)_) of plasmalemmal ionic currents in excitable cells remains largely unresolved.

The KCNQ (K_V_7) family of K^+^ channels consists of five members, designated KCNQ1-KCNQ5 (K_V_7.1–K_V_7.5). Among these, KCNQ2, KCNQ3, and KCNQ5 encode the principal subunits of K_V_7.2, K_V_7.3, and K_V_7.5 channels, respectively, which are broadly expressed in both nervous and endocrine tissues. Notably, heteromeric KCNQ2/KCNQ3 channels closely replicate the native M-type K^+^ current (*I*_K(M)_), sharing its biophysical properties and sensitivity to pharmacological inhibitors such as linopirdine. The designation “M-type” reflects the current’s regulation by muscarinic acetylcholine receptors [27,28,29]. The magnitude of these currents can widely regulate membrane excitability in varying types of excitable cells that include endocrine cells [28,29,30]. Once activated by membrane depolarization, they exhibit slow activation and deactivation kinetics [31,32,33,34]. It has also been shown that by enhancing Na^+^-current recovery, the *I*_K(M)_ amplitude induced by high frequency stimulation can expedite action potential (AP) firing with stable waveforms and reliable synaptic transmission [34,35]. Moreover, pharmacological targeting of *I*_K(M)_ (or KCNQ/K_V_7 channel-mediated currents) has been recently recognized as a potential adjunctive strategy for a range of neurological disorders characterized by neuronal hyperexcitability, including cognitive dysfunction, epilepsy, major depression, and neuropathic pain [29,35,36,37].

Building on these considerations, the present study investigated whether and how corylin modulates the magnitude, gating kinetics, and Hys_(V)_ behavior of *I*_K(M)_ in pituitary tumor (GH_3_) cells. Importantly, our findings demonstrate that, beyond its previously described anti-inflammatory, antineoplastic, and antioxidative actions, corylin can interact with K_M_ (KCNQ or K_V_7) channels to enhance *I*_K(M)_ in a concentration-, time-, voltage-, and Hys_(V)_-dependent manner in excitable cells such as GH_3_ lactotrophs.

## 2. Results

### 2.1. Stimulatory Effect of Corylin on M-Type K^+^ Current (I_K(M)_) Recorded from Pituitary GH_3_ Cells

**Figure 1:**
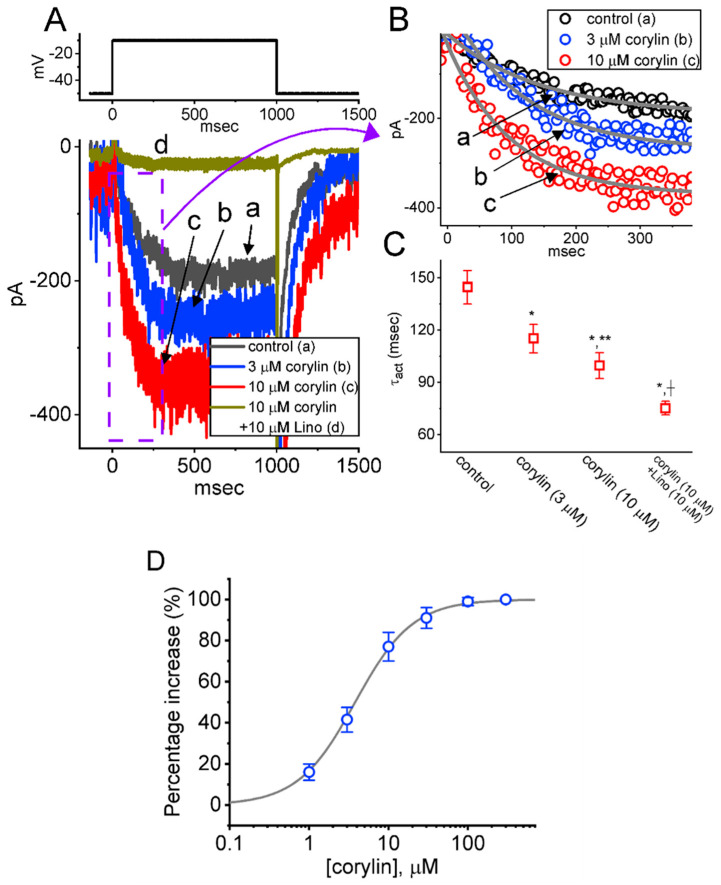
Effect of corylin on M-type K^+^ current (*I*_K(M)_) recorded from pituitary tumor (GH_3_) cells. In these experiments, we used a high-K^+^, Ca^2+^-free solution as a bathing solution, and the recording electrode was filled with a K^+^-containing solution. (**A**) Superimposed current traces acquired in the control period (i.e., corylin was not present, a, black color) and during cell exposure to 3 μM corylin (b, blue color), 10 μM corylin (c, red color), or 10 μM corylin plus 10 μM linopirdine (Lino) (d, brown color). The top part indicates the voltage-clamp protocol applied (i.e., 1-s depolarizing step from −50 to −10 mV). (**B**) Enhancing effect of corylin on the activation time course of *I*_K(M)_. The time course of *I*_K(M)_ activation in the absence (a, open black circles) and in the presence of 3 μM corylin (b, open blue circles) or 10 μM corylin (c, open red circles) was fitted using a single-exponential function (gray lines). The current trajectory labeled “d” in (**A**) is not shown. The current traces in (**B**) were an expanded record from the purple dashed box of (**A**). (**C**) Scatter graph summarizing effects of corylin (3 or 10 μM) and corylin (10 μM) plus linopirdine (10 μM, Lino) on the value of activation time constant (τ_act_) of *I*_K(M)_ in GH_3_ cells (mean ± SEM; *n* = 8 for each point). The *I*_K(M)_ was evoked by the depolarizing command voltage pulse to −10 mV for a duration of 1 s from a holding potential of −50 mV. The statistical analyses were performed using ANOVA-1, *p* < 0.05, followed by a post hoc Fisher’s LSD test, *p* < 0.05. * Significantly different from control (*p* < 0.05), ** significantly different from corylin (3 μM)-alone group (*p* < 0.05), and † significantly different from corylin (10 μM)-alone group (*p* < 0.05). (**D**) Concentration-dependent effect of corylin (1–300 μM) on the percentage increase in *I*_K(M)_ amplitude (mean ± SEM; *n* = 8 for each point). The smooth gray line represents the best fit to the data points with a modified Hill equation, as mentioned in Section 4. The EC_50_ and Hill coefficient for corylin-stimulated *I*_K(M)_ were 3.8 μM and 1.2, respectively.

In the initial set of experiments, we examined whether *I*_K(M)_ in GH_3_ cells can be modified by corylin. Cells were bathed in a high-K^+^, Ca^2+^-free solution containing 1 μM tetrodotoxin (TTX) and 0.5 mM CdCl_2_, while the recording pipette was filled with a K^+^-based internal solution. After establishing whole-cell current recordings, a 1 s depolarizing voltage pulse from −50 to −10 mV was applied to evoke inward *I*_K(M)_, which displayed a slowly activating, non-inactivating time course, consistent with previous reports [26,31,32,34,38,39,40]. However, of additional interest, as cells were exposed to corylin, the amplitude of *I*_K(M)_ activated by long-lasting step depolarization progressively increased (Figure 1A). For example, during exposure to 10 μM corylin, *I*_K(M)_ increased from 194 ± 25 to 348 ± 31 pA (*n* = 8, paired *t*-test, *p* = 0.018). Following the removal of corylin, the current amplitude returned to 202 ± 26 pA (*n* = 8). Concurrently, the value of activation time constant (τ_act_) of *I*_K(M)_ became shortened, as evidenced by a significant reduction in τ_act_ from 144.6 ± 9.6 to 99.7 ± 7.4 ms (*n* = 8, paired *t*-test, *p* = 0.014) by adding 10 μM corylin (Figure 1B,C). Furthermore, as cells were continually exposed to 10 μM corylin, the subsequent addition of 10 μM linopirdine could attenuate current amplitude as well as decrease τ_act_ value effectively (Figure 1C). Linopirdine was reported to be an inhibitor of *I*_K(M)_ [31,34,40,41]. These results suggest that corylin is capable of modulating the activation kinetics of *I*_K(M)_.

We subsequently established the concentration-dependent relationship of corylin-induced stimulation of *I*_K(M)_ and the findings are presented in Figure 1D. Notably, this compound can increase the amplitude of *I*_K(M)_ in a concentration-dependent manner. Based on a least-squares fit to the modified equation (as indicated in Section 4), the data yielded a half-maximal effective concentration (EC_50_) for *I*_K(M)_ stimulation of 3.8 μM and a Hill coefficient of 1.2. The results lead us to indicate that the addition of corylin would exert a stimulatory effect on *I*_K(M)_ in these cells.

### 2.2. Effect of Corylin on the Steady-State Current Versus Voltage (I-V) Relation and Activation Curve of I_K(M)_

**Figure 2:**
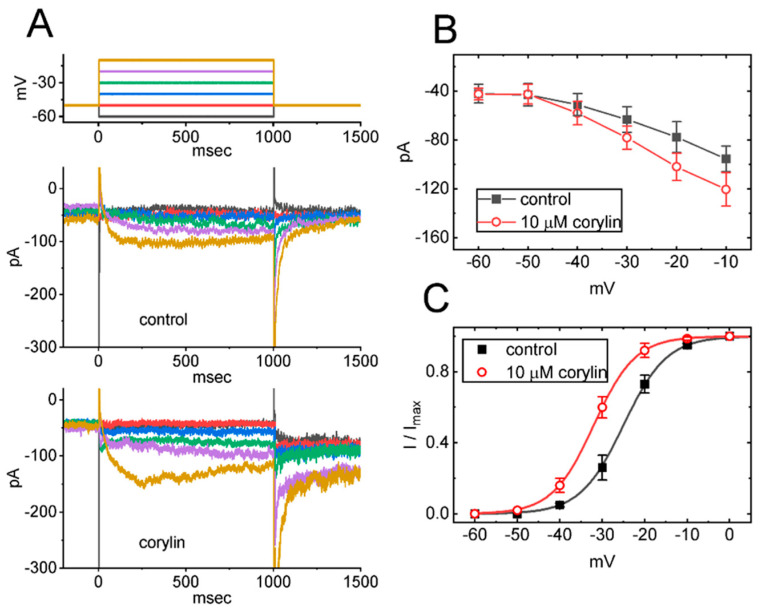
Effect of corylin on the steady-state current versus voltage (*I*-*V*) relationship (**A**) and activation curve (**B**) of *I*_K(M)_ present in GH_3_ cells. (**A**) Representative current traces acquired in the control period (i.e., corylin was not present, **upper**) and during cell exposure to 10 μM corylin (**lower**). The top part indicates the voltage-clamp protocol applied, and the voltage shown in different colors corresponds to the current trace evoked by the voltage at the same color. (**B**) Mean *I*-*V* relationship of *I*_K(M)_ acquired in the control (filled black squares) and during exposure to 10 μM corylin (open red circles) (mean ± SEM; *n* = 8 for each point). (**C**) Mean relationship of the relative current amplitude (*I*/*I*_max_) versus membrane potential of *I*_K(M)_ (i.e., the steady-state activation curve of the current) (mean ± SEM; *n* = 8 for each point). ■: control; **○**: corylin (10 μM).

Next, we wanted to test if *I*_K(M)_ activated by different levels of membrane potential can be altered by the existence of corylin. As illustrated in Figure 2A,B, when a series of voltage steps ranging from −60 to −10 mV was applied to the test cells from a holding potential of −50 mV, the absolute amplitude of *I*_K(M)_ increased progressively, particularly at membrane potentials more depolarized to −40 mV. In addition, the quasi-steady-state activation curve of *I*_K(M)_, in the absence and presence of corylin, was constructed, and the results are presented in Figure 2C. The relationship between membrane potential and normalized *I*_K(M)_ amplitude was also fitted with a Boltzmann function (see Section 4), and the fit was evaluated using goodness-of-fit analysis. In the control (i.e., corylin was not present), *V*_1/2_ = −25.1 ± 1.5 mV and *q* = 4.9 ± 0.2 *e* (*n* = 8), while during cell exposure to 10 μM corylin, *V*_1/2_ = −31.8 ± 1.7 mV and *q* = 5.1 ± 0.2 *e* (*n* = 8). Moreover, corylin increased the absolute amplitude of the deactivating tail *I*_K(M)_. For instance, during membrane depolarization from −50 to −10 mV, the tail *I*_K(M)_ measured upon repolarization to −80 mV increased from 252 ± 14 to 321 ± 19 pA (*n* = 8, paired *t*-test, *p* = 0.021). The results indicated that, in the presence of corylin, the quasi-steady-state *I*_K(M)_ activation curve was shifted leftward (toward more hyperpolarized potentials) by approximately 7 mV in the presence of corylin, without any noticeable change in the gating charge associated with channel activation.

### 2.3. Corylin’s Effect on I_K(M)_ Amplitude Evoked During a Train of Depolarizing Command Voltages in GH_3_ Cells

**Figure 3:**
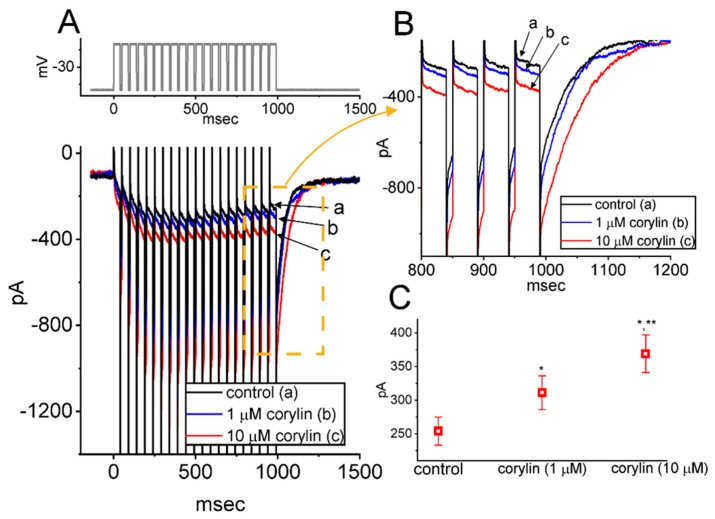
Stimulatory effect of corylin on *I*_K(M)_ amplitude induced during a 20 Hz train of depolarizing pulses in GH_3_ cells. The train was specifically designed to consist of 20 40 ms pulses separated by 10 ms intervals at −50 mV for a duration of 1 s, and through digital-to-analog conversion, it was imposed over the tested cell. (**A**) Representative current traces acquired in the control period (a, black color) and during cell exposure to 1 μM corylin (b, blue color) or 10 μM corylin (c, red color). The voltage-clamp protocol is indicated in the top part. To provide high resolution, current traces in (**B**) denote an expanded record from the dashed yellow box in (**A**). (**C**) Summary graph demonstrating the effect of corylin (1 and 10 μM) on the *I*_K(M)_ amplitude in response to a train of depolarizing command voltage from −50 to −10 mV (mean ± SEM; *n* = 7 for each point). Current amplitude was measured at the end of each train of depolarizing pulses. Of note, cell exposure to corylin produces an increase in *I*_K(M)_ amplitude activated by a train of pulses. The statistical analyses were performed using ANOVA-1, *p* < 0.05, followed by a post hoc Fisher’s LSD test, *p* < 0.05. * Significantly different from control (*p* < 0.05), and ** significantly different from the corylin (1 μM)-alone group (*p* < 0.05).

Recent work has demonstrated the effectiveness of the train of depolarizing pulses in modifying the *I*_K(M)_ magnitude [26,34,35]. For this reason, we continued to explore whether the corylin-mediated stimulation of *I*_K(M)_ in these cells can be modified during pulse-train stimulation. In this set of whole-cell current measurements, we applied a 20 Hz train of depolarizing pulses from −50 to −10 mV to the tested cell. As demonstrated in Figure 3A,B, the current activation and deactivation evoked by responding to such pulse-train stimulation were robustly observed. Furthermore, exposure of cells to corylin increased *I*_K(M)_ during a train of depolarizing pulses (Figure 3C). Concomitantly, the time course of current activation became faster in the presence of corylin. For instance, exposure to 10 μM corylin significantly increased the absolute amplitude of *I*_K(M)_ measured at the end of pulse-train stimulation, rising from 254 ± 21 to 369 ± 28 pA (*n* = 7, paired *t*-test, *p* = 0.018). In parallel, the amplitude of deactivating *I*_K(M)_ was markedly elevated from 623 ± 43 to 983 pA (*n* = 7, paired *t*-test, *p* = 0.015). Additionally, the τ_act_ value for *I*_K(M)_ during a train of command voltages was measurably shortened to 53 ± 9 ms (*n* = 7, paired *t*-test, *p* = 0.019) from a control value of 114 ± 14 ms (*n* = 7). Therefore, exposure to corylin produced a considerable increase in the amplitude of *I*_K(M)_, along with a reduction in the τ_act_ value during a train of depolarizing pulses.

### 2.4. Augmentation of the Strength in Voltage-Dependent Hysteresis (Hys_(V)_) of I_K(M)_ Caused by Corylin

**Figure 4:**
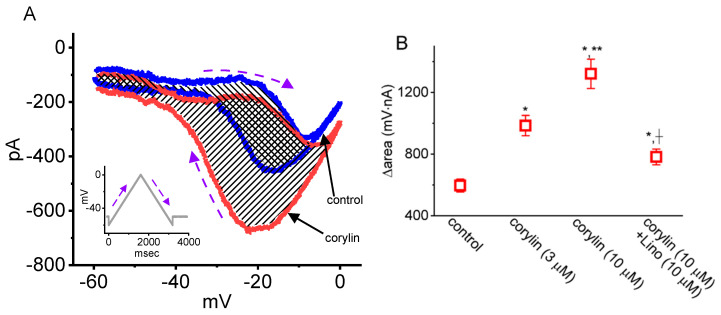
Stimulatory effect of corylin on the strength of voltage-dependent hysteresis (Hys_(V)_) activated by upright isosceles-triangular ramp pulse (V_ramp_). In this series of whole-cell current recordings, the tested cell was voltage-clamped at −50 mV, and a triangular V_ramp_ with a duration of 3.6 s (ramp speed ± 16.7 mV/sec) was applied to elicit instantaneous *I*_K(M)_. Under these conditions, whole-cell *I*_K(M)_ was robustly evoked during the forward (ascending from −60 to 0 mV) and backward (descending from 0 to −60 mV) limbs of V_ramp_ commands. (**A**) Relationship between *I*_K(M)_ and membrane potential (i.e., Hys_(V)_ behavior) obtained under the control conditions (blue trace) and during exposure to 10 μM corylin (red trace). The inset illustrates the voltage-clamp pulse protocol, and the dashed purple arrows in both the inset and panel (**A**) indicate the direction of the current trajectory over time. A clockwise Hys_(V)_ loop evoked by the double V_ramp_ protocol (duration 3.2 s; ramp speed ±16.7 mV/s) was clearly observed. The application of corylin (10 μM) increased the strength of the V_ramp_-induced Hys_(V)_, as indicated by the shaded region. The left hatched region (outlined by the blue lines) was obtained under control conditions. The right hatched region (outlined by the red lines) was acquired in the presence of corylin. The cross-hatched area represents the overlap between the two regions. (**B**) Scatter plot summarizing changes in the hysteresis area (∆area) of the V_ramp_-induced Hys_(V)_ loop measured during exposure to 3 or 10 μM corylin, as well as 10 μM corylin in the presence of 10 μM linopirdine (Lino) (mean ± SEM; *n* = 8 for each point). The ∆area of the Hys_(V)_ loop was calculated as the area enclosed by the current traces generated during the forward (upsloping) and backward (downsloping) limbs of the triangular V_ramp_. The statistical analyses were performed using ANOVA-1, *p* < 0.05, followed by a post hoc Fisher’s LSD test, *p* < 0.05. * Significantly different from control (*p* < 0.05), ** significantly different from corylin (3 μM)-alone group (*p* < 0.05), and † significantly different from corylin (10 μM)-alone group (*p* < 0.05).

It has been demonstrated that V_ramp_-induced Hys_(V)_ of *I*_K(M)_ could contribute to AP firing in various types of excitable cells [26,32,34]. Hys_(V)_ refers to a pronounced lag in current magnitude when the membrane potential is linearly ramped in the opposite direction. Accordingly, the experiments were designed to determine whether V_ramp_-induced Hys_(V)_ was functionally active in GH_3_ cells and to evaluate how cell exposure to corylin may influence the Hys_(V)_’s strength in *I*_K(M)_. In whole-cell voltage-clamp recordings, the cell was held at −50 mV, after which a series of double triangular V_ramp_ pulses (ranging between −60 and 0 mV, 3.6 s in duration, delivered at 0.05 Hz) was applied via a digital-to-analog conversion. In accordance with previous observations [26,32,34], when a double V_ramp_ was applied to the examined cells, the current amplitude at a given membrane potential evoked during the ascending (forward or upsloping) limb of V_ramp_ was markedly smaller than that measured at the same voltage during the descending (backward or downsloping) limb of the voltage (Figure 4A). For example, in the control period (i.e., in the absence of corylin), the absolute amplitude of *I*_K(M)_ at −20 mV differed significantly between the ascending (upsloping) and descending (downsloping) phases of V_ramp_, measuring 198 ± 22 and 418 ± 28 pA (*n* = 8, paired *t*-test, *p* = 0.011). These findings strongly indicate the presence of *I*_K(M)_’s Hys_(V)_ in response to an upright isosceles-triangular V_ramp_ in GH_3_ cells [34].

Of additional notice, as GH_3_ cells were continually exposed to corylin, the Hys_(V)_ strength of *I*_K(M)_ became accentuated. For example, in the presence of 10 μM corylin, *I*_K(M)_ amplitude (in absolute value) at the level of −20 mV evoked during the ascending or descending end of V_ramp_ became measurably raised to 218 ± 23 pA (*n* = 8, paired *t*-test, *p* = 0.014) or 682 ± 38 pA (*n* = 8, paired *t*-test, *p* = 0.013), respectively. It was therefore observed that the current magnitude at the descending end of V_ramp_ increased more significantly than that at the ascending phase of V_ramp_. We then quantify the strength of the Hys_(V)_ loop by estimating the total area (∆area, shaded region), which encircles the current amplitude between the ascending and descending ends of V_ramp_. The experimental data were compiled and are summarized in Figure 4B. These observations clearly demonstrate that corylin (3 or 10 μM) increased Hys_(V)_’s ∆area of *I*_K(M)_ in GH_3_ cells. Additionally, during the continued presence of corylin (10 μM), the subsequent addition of linopirdine (10 μM) was able to attenuate the corylin-increased ∆area of *I*_K(M)_ evoked during isosceles-triangular V_ramp_. Therefore, these findings suggest that the presence of corylin increases *I*_K(M)_ in a concentration- and Hys_(V)_-dependent manner in these cells.

### 2.5. Comparison Among Effects of Corylin, Corylin Plus Carvedilol (Carv), Corylin Plus Iberiotoxin (Iber), 17β-Estradiol, and Corylin Plus Dapagliflozin (Dapa) on I_K(M)_ Amplitude Observed in GH_3_ Cells

**Figure 5:**
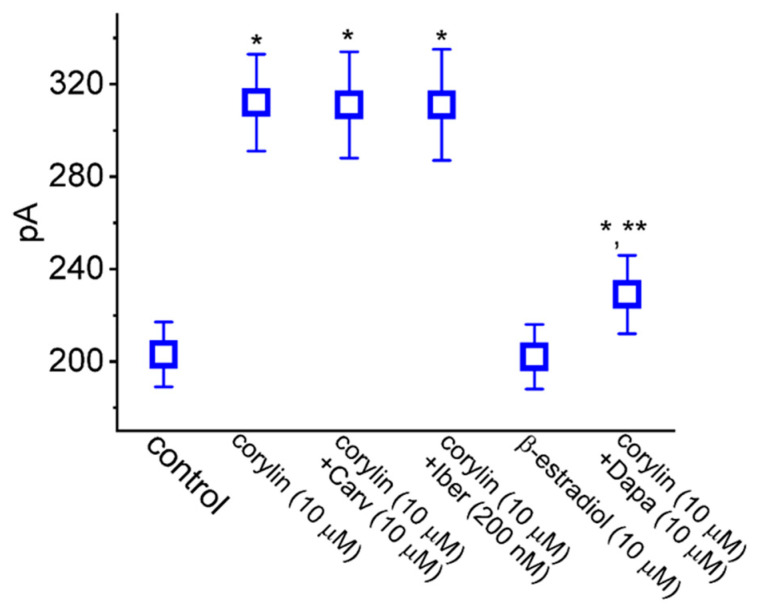
Summary scatter graph demonstrating effects of corylin, corylin plus carvedilol (Carv), corylin plus iberiotoxin (Iber), 17β-estradiol (β-estradiol), and corylin plus dapagliflozin (Dapa) on *I*_K(M)_ amplitude in GH_3_ cells. In these experiments, we placed cells in a high-K^+^, Ca^2+^-free solution and the recording pipette was filled with a K^+^-containing solution. In absolute value, current amplitudes during cell exposure to different tested compounds were measured at the end of a 1 s depolarizing pulse from −50 to −10 mV. Each data point represents the mean ± SEM (*n* = 8). The statistical analyses were performed using ANOVA-1, *p* < 0.05, followed by a post hoc Fisher’s LSD test, *p* < 0.05. * Significantly different from control (*p* < 0.05) and ** significantly different from corylin (10 mM) alone (*p* < 0.05).

Recent reports have demonstrated the ability of corylin to bind to β_3_-adrenergic receptors in adipocytes [11,18,42]. The induction of osteoblastic differentiation caused by corylin was mediated through its binding to and interaction with estrogen receptors [18,42]. Pituitary lactotrophs have been previously reported to express the activity of estrogen receptors [43]. It would thus be important to examine whether corylin-stimulated *I*_K(M)_ presented herein could be a result of its binding to β-adrenergic or estrogen receptors. Under our experimental conditions, as cells were continually exposed to 10 μM corylin, the addition of neither carvedilol (Carv, 10 μM) nor iberiotoxin (200 nM) was able to have any modifications on corylin-stimulated *I*_K(M)_, as summarized in Figure 5. Carvedilol, a non-selective β-adrenergic blocker, was shown previously to antagonize the activity of β_3_-adrenergic receptors expressed in cardiac tissue [44]. It is thus thought that carvedilol might block the *I*_K(M)_-stimulating effects of corylin via β-adrenergic receptors. Iberiotoxin was an inhibitor of large-conductance Ca^2+^-activated K^+^ channels [45]. Moreover, the addition of 17β-estradiol (10 μM) was found to have no effect on *I*_K(M)_. In the continued presence of 10 μM corylin, the subsequent addition of dapagliflozin (10 μM) could attenuate the corylin-induced stimulation of *I*_K(M)_. Dapagliflozin (Dapa), an inhibitor of Na^+^-dependent glucose co-transporters, has been reported to suppress *I*_K(M)_ directly [39]. Based on the present observations, it is therefore unlikely that the corylin-stimulated *I*_K(M)_ observed in GH_3_ cells results primarily from its binding to β-adrenergic or estrogen receptors.

### 2.6. Effect of Corylin on M-Type K^+^ (K_M_) Channels Recorded from GH_3_ Cells

**Figure 6:**
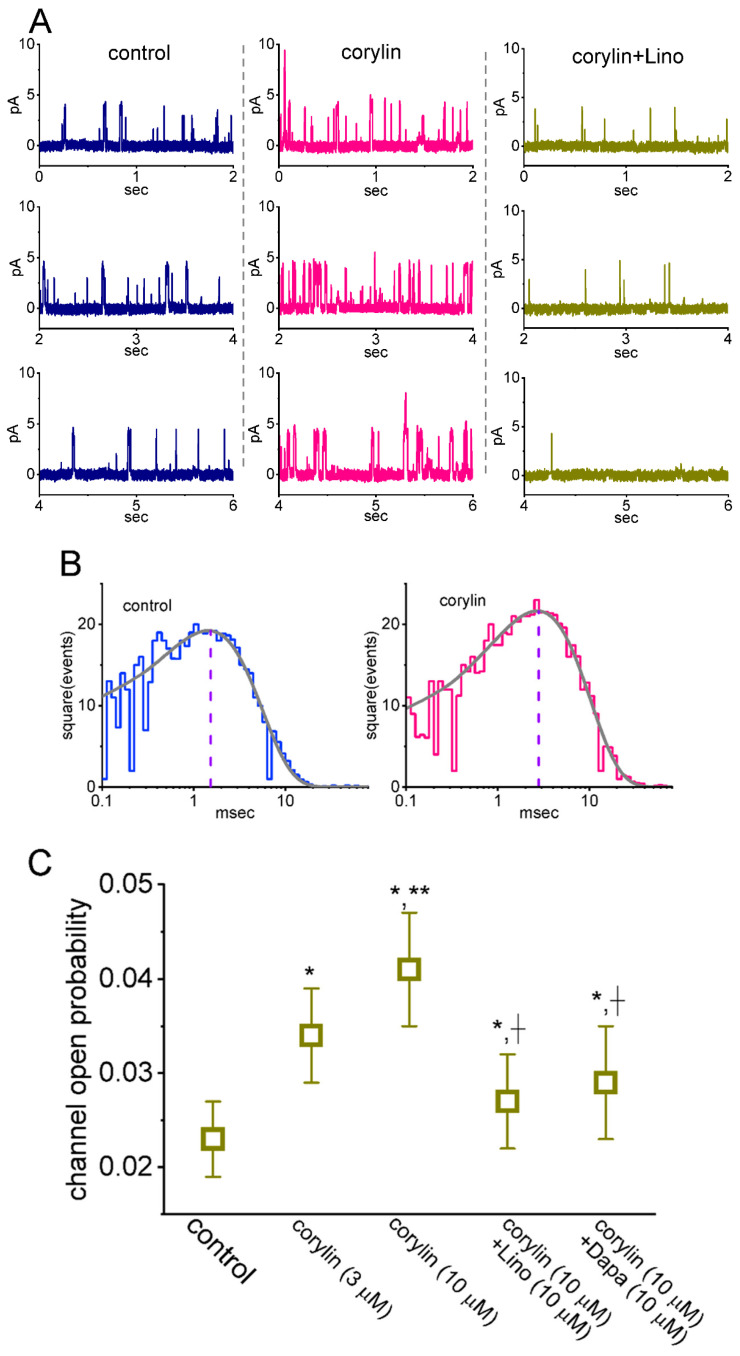
Effect of corylin on the activity of M-type K^+^ (K_M_) channels recorded from GH_3_ cells. This set of cell-attached recordings was made in cells maintained in a high-K^+^, Ca^2+^-free solution, and the recording pipette was filled with a low-K^+^ (5.4 mM) solution. (**A**) Representative channel activity acquired in the control period (**left**, blue color), after the addition of 10 μM corylin (**middle**, red color), and after the addition of 10 μM corylin plus 10 μM linopirdine (Lino) (**right**, brown color). The single-channel events were measured as the tested cell was voltage-clamped at +20 mV relative to the bath. The upward deflection indicates the opening event of the K_M_ channel, which occurs with rapid open–closed transitions. (**B**) Effect of corylin on the mean open time of K_M_ channels. In the control (**left**), data were obtained from the measurements of 243 channel openings, with a total recording time of 2 min, whereas in the presence of 10 μM corylin (**right**), data were from 278 channel openings, with a total recording time of 1 min. Of note, the x- and y-axes indicate the square root of the event number and the logarithm of open time (ms), respectively. In each lifetime distribution, the continuous line represents the optimal fit to a single-exponential function, while the vertical dashed line marks the corresponding constant, indicating the mean open time. (**C**) Summary scatter graph demonstrating the effects of corylin (3 or 10 μM), corylin plus linopirdine (Lino), and corylin plus dapagliflozin (Dapa) on channel open probability (mean ± SEM; *n* = 8 for each point). Channel activity was measured at +20 mV relative to the bath. The statistical analyses were performed using ANOVA-1, *p* < 0.05, followed by a post hoc Fisher’s LSD test, *p* < 0.05. * Significantly different from control (paired *t*-test, *p* < 0.05), ** significantly different from corylin (3 μM)-alone group (*p* < 0.05), and † significantly different from corylin (10 μM)-alone group (*p* < 0.05).

The corylin-induced enhancement of whole-cell *I*_K(M)_ may result from several mechanisms. Specifically, alterations in channel open probability, single-channel conductance, gating kinetics (e.g., mean open time), or a combination of these factors could underlie the observed stimulation of *I*_K(M)_. To further investigate single-channel activity in K_M_ channels in the presence or absence of corylin, additional measurements were performed. In these cell-attached recordings, cells were maintained in a high-K^+^, Ca^2+^-free solution, while the recording electrode was filled with a low-K^+^ (5.4 mM) solution. As illustrated in Figure 6, when the tested cell was voltage-clamped at +20 mV relative to the bath, the activity of single K_M_ channels was robustly observed [31,39,40]. When corylin was applied to the bath, the channel open probability was progressively increased. In the continued presence of corylin, further addition of linopirdine attenuated a corylin-induced increase in channel activity. For example, the presence of 10 μM corylin conceivably increased the probability of K_M_-channel openings from 0.023 ± 0.004 to 0.041 ± 0.006 (*n* = 8, paired *t*-test, *p* = 0.011); however, no modification in the single-channel amplitude was found (3.3 ± 0.2 pA [control] versus 3.4 ± 0.3 pA [in the presence of corylin]; *n* = 8, paired *t*-test, *p* = 0.091). After the washout of corylin, channel activity recovered to 0.024 ± 0.006 (*n* = 7, paired *t*-test, *p* = 0.021).

We further examined and analyzed the kinetic properties of K_M_ channels obtained with or without the addition of corylin. As demonstrated in Figure 6B, the distribution of open durations was least-squares fitted by a single exponential. The mean open time of K_M_ channels rose from 1.5 ± 0.2 to 2.8 ± 0.3 ms (*n* = 8, paired *t*-test, *p* = 0.018). The corylin-induced modulation of K_M_-channel activity likely reflects an increase in channel open duration rather than any change in single-channel amplitude.

Moreover, in the continued presence of 10 μM corylin, further addition of linopirdine (10 μM) or dapagliflozin (10 μM) was able to attenuate corylin-enhanced channel activity effectively (Figure 6C). Linopirdine can suppress the activity of K_M_ channels, while dapagliflozin, known to be an inhibitor of Na^+^-dependent glucose co-transporter, was reported to inhibit *I*_K(M)_ amplitude [34,39]. Therefore, corylin-stimulated *I*_K(M)_ could be reasonably explained by the increased channel open probability as well as by its prolongation in the mean open time of K_M_ channels.

### 2.7. Effect of Corylin on Erg-Mediated K^+^ Current (I_K(erg)_) Recorded from GH_3_ Cells

**Figure 7:**
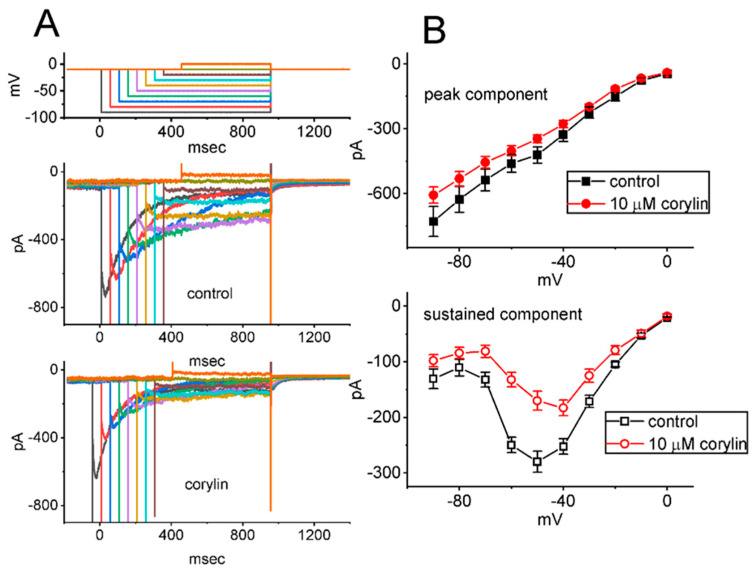
Inhibitory effect of corylin on *erg*-mediated K^+^ current (*I*_K(erg)_) residing in GH_3_ cells. In these experiments, cells were suspended in a high-K^+^, Ca^2+^-free solution containing 1 μM TTX and 0.5 mM CdCl_2_, and the recording pipette used was filled with K^+^-enriched solution. After establishing the whole-cell configuration, the tested cell was held at −10 mV, and a series of rectangular voltage pulses ranging from −90 to 0 mV in 10-mV increments was applied. (**A**) Superimposed current traces acquired in the absence (**upper part**) and presence (**lower part**) of 10 μM corylin. The uppermost graph in (**A**) denotes the voltage-clamp protocol applied to the examined cell. The voltage traces shown in different colors correspond with current ones evoked by the same levels of membrane potential. (**B**) Average *I*-*V* relationship of peak (**upper**, filled symbols) and sustained (**lower**, open symbols) components of deactivating *I*_K(erg)_ obtained in the absence (black squares) or presence (red circles) of 10 μM corylin (mean ± SEM; *n* = 8 for each point). Current amplitudes were measured at the start (peak component) and end pulse (sustained component) of various command voltage steps. Current amplitudes measured between −50 and −80 mV exhibit an inwardly rectifying property of the absolute *I*_K(erg)_. Of note, the *I*_K(erg)_ in GH_3_ cells was subjected to mild inhibition by the existence of corylin (10 μM).

In another set of measurements, we attempted to examine if another type of K^+^ current (e.g., *I*_K(erg)_) would be sensitive to any perturbations by the presence of corylin. To measure *I*_K(erg)_ [39,46], we put GH_3_ cells in a high-K^+^, Ca^2+^-free solution, and we then filled up the recording pipette with a K^+^-enriched solution. As the whole-cell configuration was established, we held the examined cell at −10 mV and a series of command voltage steps ranging between −90 and 0 mV with varying durations was imposed on it. As shown in Figure 7, under cell exposure to 10 μM corylin, the *I*_K(erg)_ amplitudes measured throughout the entire voltage-clamp voltages imposed were conceivably reduced. For example, upon exposure to 10 μM corylin, the absolute peak amplitude of deactivating *I*_K(M)_, elicited by membrane hyperpolarization from −10 to −90 mV, decreased from 729 ± 70 to 607 ± 38 pA (*n* = 8, paired *t*-test, *p* = 0.021). After washout of the compound, the current amplitude was returned to 724 ± 68 pA (*n* = 8). Therefore, unlike its stimulation of *I*_K(M)_, the *I*_K(erg)_ observed in GH_3_ cells [30,39,46] was susceptible to being mildly inhibited by adding corylin.

### 2.8. Docking Results of the Molecular Interactions Between Corylin and the KCNQ2 or KCNH2 Channel

**Figure 8:**
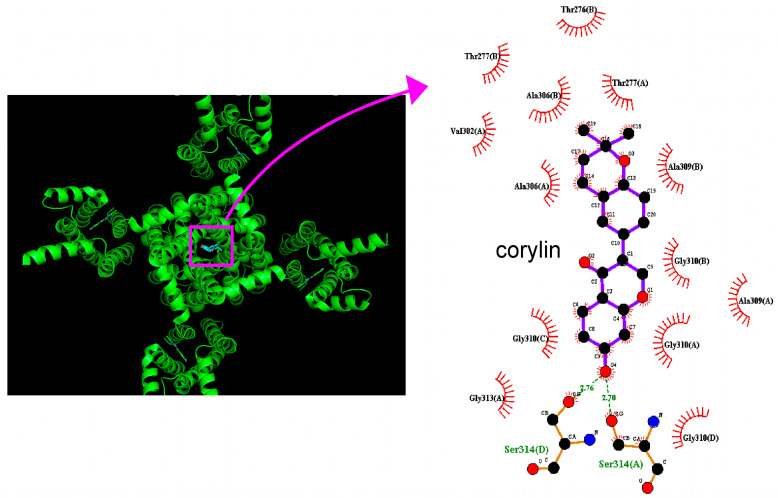
Docking results of the KCNQ2 channel and corylin. Protein structure of the KCNQ2 channel was acquired from PDB (PDB ID: 7CR1), while the three-dimensional structure of corylin is from PubChem (compound CID: 5316097). The structure of KCNQ2 auto-docked with the corylin molecule was made through PyRx (http://pyrx.sourceforge.io/, accessed on 18 March 2026). The diagram of interaction between KCNQ2 and the corylin molecule was generated by LigPlot^+^ v.2.3 (https://www.ebi.ac.uk/thornton-srv/software/LigPlus/download.html, accessed on 18 March 2026). The (**right**) panel shows an enlarged view of the region highlighted by the pink box with the curved arrow in the (**left**) panel. Notably, in this and the subsequent figures, red arcs with spokes directed toward the ligand (corylin) denote hydrophobic interactions between the protein and the corylin molecule, whereas green dotted lines indicate hydrogen bonds. In the central part of the right panel of this and the next figures, the chemical structure of corylin is shown. The parentheses following the amino acid indicate the chain identifier.

In this study, we examined how the protein of KCNQ2 could be auto-docked by corylin through PyRx 0.8 software (https://pyrx.sourceforge.io, accessed on 18 March 2026). The predicted binding sites of corylin are presented in Figure 8. It needs to be emphasized that corylin can form hydrophobic contact with several amino acid residues, including Thr276, Thr277, Val302, Ala306, Ala309, Gly310 and Gly313, while it forms hydrogen bonds with residue Ser 314 with distances of 2.70 and 2.76 Å, and that the estimated binding affinity among this interaction was −8.4 kcal/mol. Based on the experimental findings described above, the docking analysis suggests that corylin may interact with the intracellular domain located near the transmembrane S6 segment of the channel. Such binding implies that the functional activity of K_M_ (KCNQ or K_V_7) channels in excitable cells could be subject to modulation by corylin or by its structurally related analogs [26,27].

**Figure 9:**
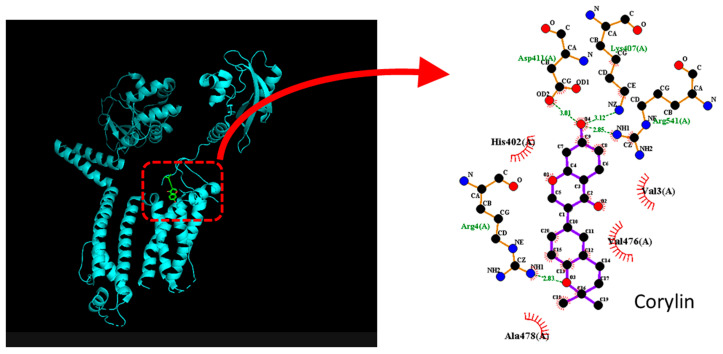
Docking results of HERG (KCNH2) and corylin. The protein structure of KCNH2 was acquired from PDB (PDB ID:5VA1) and the three-dimensional structure of corylin is from PubChem (Compound CID: 5316097). The structure of KCNH2 was optimally docked with corylin using PyRx, as highlighted in the red dashed box on the left and indicated by the red curved arrow.

Because corylin can inhibit the amplitude of *I*_K_(erg)_,_ the KCNH2 (HERG, human *ether-à-go-go*-related gene) protein was further docked with corylin using PyRx software. The predicted binding sides of corylin on this channel protein are illustrated in Figure 9. This compound was observed to establish hydrophobic interactions with several residues, including Val3(A), His402(A), Val476(A), and Ala478(C). The corylin molecule can form hydrogen bonds with residues Arg4(A), Lys407(A), Asp411(A), and Arg541(A), with bond lengths of 2.81, 3.12, 3.01, and 2.85 Å, respectively. The results estimate a strong binding affinity of −7.8 kcal/mol. The predicted interaction thus suggests that corylin-mediated inhibition of *I*_K(erg)_ in GH_3_ cells is due to its binding to KCNH2 channels. However, it needs to be mentioned that the proposed interaction with KCNQ2 and KCNH2 channels is hypothetical and based primarily on in silico docking results, rather than direct functional validation. Interactions with other subtypes, such as KCNQ3 and KCNQ5 channels, also cannot be excluded.

## 3. Discussion

The striking findings demonstrated in this work are that corylin, a bioactive flavonoid currently recognized as a potential life-prolonging agent [47], produces a stimulatory action on *I*_K(M)_ in a concentration-, voltage-, and Hys_(V)_-dependent fashion in GH_3_ lactotrophs. A leftward shift in the steady-state activation curve of *I*_K(M)_ was observed in the presence of this compound, with no change in the gating charge of the curve. Cell exposure to it can elevate the probability of K_M_ channels that would be open, in combination with a measurable lengthening in the mean open time of the channel. However, the *I*_K(erg)_ in GH_3_ cells was slightly suppressed by the presence of corylin. Docking analysis revealed specific atomic-level interactions between the corylin molecule and the KCNQ2 or KCNH2 channel structure.

The magnitude of Na^+^ currents can rapidly decline in an exponential manner during high-frequency stimulation, as previously demonstrated in GH_3_ cells [48]. However, it needs to be emphasized that because of its slow activation and deactivation kinetics, the *I*_K(M)_ amplitude can progressively rise during the repetitive firing of APs. During high-frequency stimulation, the accumulation of *I*_K(M)_ is allowed to hyperpolarize the afterpotential and hence speed the recovery of Na^+^ channels from inactivation. As a corollary, the augmentation of *I*_K(M)_ magnitude caused by corylin during high-frequency activity is of particular significance and thus capable of facilitating the firing of neuronal APs with a stable waveform and high-fidelity synaptic signaling [26,34,35].

Like the Hys_(V)_ behavior in solar cells [34,49], the current investigations clearly observed the appearance of the overall behavior in *I*_K(M)_’s Hys_(V)_ evoked by a long-lasting upright isosceles-triangular V_ramp_ [30,32,39]. That is, the magnitude of these instantaneous currents measured between ascending and descending ends of double V_ramp_ turned out to be strikingly distinguishable. Alternatively, as the membrane potential becomes depolarized (i.e., upward ramp of triangular V_ramp_), the voltage dependence of K_M_ channels may shift the mode of Hys_(V)_ to one that occurs at less negative potentials with smaller current magnitude, leading to a minor effect on the rising phase of AP. However, as the membrane potential is hyperpolarized (i.e., during the downward limb of V_ramp_ or repolarizing phase of AP), the voltage dependence of *I*_K(M)_ activation would switch to more hyperpolarized voltages with a higher current magnitude, thereby having the tendency to increase membrane repolarization as well as to increase the recovery of Na^+^ currents. Furthermore, these findings revealed that the triangular V_ramp_-induced *I*_K(M)_ exhibited a pronounced voltage-dependent Hys_(V)_, and the magnitude of these Hys_(V)_ loops was further enhanced by the addition of corylin. In other words, the ∆area (indicated in the shaded area of Figure 4A) of the *I*_K(M)_ loop evoked in response to long-lasing triangular V_ramp_ significantly increased following the application of corylin. As such, the existence of corylin may increase *I*_K(M)_ magnitude in a concentration- and Hys_(V)_-dependent fashion. However, further work needs to be conducted to examine if corylin-perturbed modifications in Hys_(V)_ behavior of *I*_K(M)_ are tightly linked to conformational changes or docking interactions in the voltage sensors of the K_M_ channel.

Earlier investigations have revealed the ability of corylin to bind to and then to activate β_3_-adrenergic receptors present in adipocytes [11,42]. It has also been demonstrated that corylin might interact with estrogen receptors to induce osteoblastic differentiation [18,50]. It has been noticed that estrogen receptors were expressed in pituitary lactotrophs [43]. However, in our study, during the continued exposure to corylin, further application of carvedilol, known to block β_3_ adrenoceptors in cardiac tissue, failed to have any effect on corylin-stimulated *I*_K(M)_. Moreover, the presence of 17β-estradiol alone did not cause any perturbations in *I*_K(M)_ observed in GH_3_ cells. In this scenario, it appears unlikely that under our experimental conditions, corylin-mediated stimulation of *I*_K(M)_ or K_M_-channel activity is attributed to its high-affinity binding to either β-adrenergic or estrogen receptors.

The EC_50_ of corylin required to stimulate *I*_K(M_ in GH_3_ cells was determined to be approximately 3.8 μM. This value aligns closely with the concentration ranges (1–300 μM) previously reported for its antioxidative, anti-inflammatory, and antineoplastic effects [4,7,8,9,11,12,15,16,47,51,52]. It should also be noted that the perturbations of corylin on membrane excitability may be strongly influenced by several confounding factors, including the concentration of corylin, the baseline resting potential, the firing patterns of APs, or a combination of these variables. It is, therefore, anticipated that the K_M_ channel is an important target for the action of corylin. The concentrations used to affect the magnitude, gating kinetics and Hys_(V)_ behaviors of *I*_K(M)_ presented herein would be pharmacologically significant in body fluids and tissues. The corylin molecule may have the propensity to exercise a higher affinity to the open state than to the resting (closed) state in the K_M_ channel, thereby destabilizing the open conformation, while the detailed ionic mechanism of corylin actions on ionic currents is not thoroughly understood.

The use of a high-K^+^ external solution in our whole-cell recordings was primarily intended to enhance the detectability and measurement accuracy of *I*_K(M)_. Specifically, elevating the extracellular K^+^ concentration reduces the K^+^ equilibrium potential difference and increases the driving force for inward K^+^ currents at subthreshold membrane potentials. This manipulation allows *I*_K(M)_ to be more readily resolved, particularly in GH_3_ cells, where the basal amplitude of this current can be relatively small under physiological K^+^ conditions. In addition, the adoption of symmetrical or near-symmetrical K^+^ conditions in this study is a widely used approach to enhance signal resolution and reduce interference from other ionic currents [38]. Increasing extracellular K^+^ concentration may influence channel gating kinetics, conductance, and pharmacological sensitivity. However, our primary objective was to examine the modulatory effect of corylin on *I*_K(M)_ under controlled and consistent recording conditions. Therefore, all experimental comparisons (control vs. corylin-treated) were conducted under the same high-K^+^ environment, ensuring that the relative changes observed can be attributed to the action of corylin rather than differences in ionic conditions. Nonetheless, future studies under physiological K^+^ conditions will be valuable to further validate the findings.

The use of GH_3_ tumor cells in this study could present limitations in terms of translatability to neurons and in vivo systems. The electrophysiological properties of *I*_K(M)_ and *I*_K(erg)_ in these cells have been shown to be consistent with those in neurons as well as in neuroendocrine or endocrine cells. Therefore, our findings—particularly corylin-induced stimulation of *I*_K(M)_—may have broader relevance and could be applicable to other types of electrically excitable cells.

Although the concentrations of corylin applied in this study (up to 300 μM) exceed typical physiological levels, the compound demonstrated notable activity. The EC_50_ for corylin-induced enhancement of *I*_K(M)_ in GH_3_ cells was 3.8 μM. Importantly, this stimulatory effect was influenced by membrane voltage, ongoing action potential firing, and the intrinsic strength of *I*_K(M)_ Hys_(V)_. Because our experiments were conducted at room temperature (approximately 25 °C), it remains essential to determine whether corylin produces comparable modulation of *I*_K(M)_ under true physiological conditions. Taken together, these findings indicate that corylin’s ability to stimulate *I*_K(M)_ may retain pharmacological relevance.

A previous paper [53] reported that the single-channel amplitude of K_M_ channels was lower than that observed in the present study. The reason for this discrepancy remains unclear. One possible explanation is that the single-channel conductance of K_M_ channels may vary among different tissue preparations. Our findings are consistent with those reported in earlier studies [31,38,54]. Because the single-channel conductance of KCNQ2 and KCNQ3 channels was reported to be higher than that of KCNQ4 and KCNQ5 channels, it remains to be determined whether corylin differentially regulates distinct populations of K_M_ (KCNQ/K_7_) channels.

In this study, we did not perform functional validation of the predicted binding sites on KCNQ2 or KCNH2 channels. Moreover, binding affinity values alone are insufficient to confirm channel modulation under physiological conditions. Docking results suggest a potential binding interaction but do not establish causality or physiological relevance. Complementary studies—including gene knockdown or overexpression systems to evaluate channel-specific effects, electrophysiological recording to assess channel activity, and site-directed mutagenesis to verify predicted binding residues—will be necessary and are planned for future investigation. Moreover, the use of subtype-selective blockers or modulators of KCNQ2 and KCNH2 channels to assess functional involvement is important to further elucidate their functional involvement.

## 4. Materials and Methods

### 4.1. Chemicals, Drugs, Reagents and Solutions

Corylin (IUPAC name: 3-(2,2-dimethylchromen-6-yl)-7-hydroxychromen-4-one, 53947-92-5, SCHEMBL1096083, CHEMBL1271888, C_20_H_16_O_4_, CAS: 53947-92-5; PubChem CID: 5316097) was supplied by MedChemExpress (GeneChain, Kaohsiung, Taiwan). Iberiotoxin was purchased from Alomone Labs (Asia Bioscience, Taipei, Taiwan), dapagliflozin (Dapa) was from Cayman (Ann Arbo, MI, USA), and carvedilol (Carv) was from Tocris (Union Biomed, Taipei, Taiwan), while 17β-estradiol, linopirdine (Lino), and tetrodotoxin (TTX) were from Sigma-Aldrich (Merck, Taipei, Taiwan). Corylin, carvedilol, dapagliflozin, and linopirdine were dissolved in dimethyl sulfoxide (DMSO) as a 20 mM stock solution and were thereafter diluted in extracellular solution to the final concentration achieved, while iberiotoxin was dissolved in 0.9% NaCl. For cell preparations, all culture media, horse and fetal calf sera, L-glutamine, and trypsin/EDTA were acquired from HyClone^TM^ (Thermo Fisher, Logan, UT, USA), while other chemicals or reagents were of laboratory grade and taken from standard sources.

The extracellular solution (normal Tyrode’s solution buffered with HEPES) contained the following ionic composition (in mM): NaCl 136.5, KCl 5.4, MgCl_2_ 0.53, CaCl_2_ 1.8, glucose 5.5, and HEPES 5.5, adjusted to pH 7.4 with NaOH. For the recording of macroscopic K^+^ currents (*I*_K(M)_ or *I*_K(erg)_), the pipette solution consisted of (in mM): KCl 140, MgCl_2_ 1, Na_2_ATP 4, Na_2_GTP 0.1, EGTA 0.1, and HEPES, titrated to pH 7.2 with KOH. To measure *I*_K(M)_, *I*_K(erg)_, or K_M_-channel activity, the bath solution contained a high K^+^ solution (in mM): KCl 130, NaCl 10, MgCl_2_ 3, glucose 6, and HEPES 10, adjusted to pH 7.4 with KOH. To record the activity of single K_M_ channels, the pipette solution was composed of the following (in mM): NaCl 136.5, KCl 5.4, MgCl_2_ 0.53, and HEPES-NaOH buffer 5 (pH 7.4).

### 4.2. Cell Preparations

GH_3_ pituitary tumor cells (BCRC-60015; Bioresources Collection and Research Center, Hsinchu, Taiwan) were cultured in Ham’s F-12 media supplemented with 15% horse serum (*v*/*v*), 2.5% fetal calf serum (*v*/*v*), and 2 mM L-glutamine [23,48,55]. To induce differentiation, cells were transferred to a serum-free, Ca^2+^-free medium. Under these experimental conditions, cell viability typically remained at 80–90% for up to two weeks. Cultures were maintained at 37 °C in a humidified incubator with a CO_2_/air mixture (1:19).

### 4.3. Electrophysiological Measurements

Before each experiment, GH_3_ cells were carefully dispersed with 1% trypsin/EDTA solution, and we thereafter quickly put an aliquot of cell suspension in a recording chamber mounted on the stage of a CKX-41 inverted microscope (Olympus; Yuan Yu, Taipei, Taiwan). Cells were immersed at room temperature (20–25 °C) in HEPES-buffered normal Tyrode’s solution that contained 1.8 mM CaCl_2_. When they were left to adhere to the chamber’s bottom for several minutes, the measurements were performed. Ionic currents were recorded with patch electrodes in the cell-attached or whole-cell configuration of a modified patch clamp technique, as described elsewhere [34,38,54,55]. GΩ-seals were typically formed in an all-or-none manner, leading to an improvement in signal-to-noise ratio. The recording pipette was connected to the input stage of an RK-400 (Bio-Logic, Claix, France) or an Axopatch-200B patch-clamp amplifier (Molecular Devices, Bestgen Biotech, New Taipei City, Taiwan). Patch electrodes (3–5 MΩ in bathing solution) were made from Kimax^®^-51 borosilicate capillary tubes (#34500; Merck, Taipei, Taiwan) using a two-step vertical puller (PB-7; Narishige, Taiwan Instrument, Tainan, Taiwan), and their tips were heat-polished in an MF-83 microforge (Narishige). All potentials were corrected for the liquid junction potential that would develop at the pipette tip in situations where the composition of the pipette internal solution was different from that in the bath medium. Tested compounds were applied by perfusion or added to the bath to obtain the final concentration indicated. In the experiments with corylin plus linopirdine, linopirdine was applied after the addition of corylin. When high-frequency stimuli were needed, we used an Astro-Med Grass S85X dual output pulse stimulator (Grass; Zhong Yan, Kaohsiung, Taiwan) [26,48,56].

The current and voltage signals were monitored in real time and recorded onto a laptop computer. The recorded data were low-pass filtered at 2 kHz using an FL-4 four-pole Bessel filter (Dagan, Minneapolis, MN, USA) and digitized at 10 kHz or more with a Digidata 1440A interface (Molecular Devices). The device was connected to either an RK-400 or Axopatch-200B patch-clamp amplifier, which was controlled via a universal serial bus (USB) connection using the pClamp 10.6 software (Molecular Devices). Ionic currents obtained from whole-cell and single-channel recordings were analyzed offline using pClamp 10.7, OriginPro^®^ (OriginLab; Scientific Formosa, Kaohsiung, Taiwan) and custom-written macros in Excel^®^ 2021 (Microsoft, Redmond, WA, USA) running on Windows 11. Capacitive transients following repolarization were commonly observed; therefore, the tail-deactivating K^+^ currents were measured after the capacitive currents had settled, typically between 10 and 20 ms after the end of the voltage pulse.

### 4.4. Data Analyses

To determine the concentration-dependent stimulatory effect of corylin on the amplitude of *I*_K(M)_, GH_3_ cells were bathed in a high-K^+^, Ca^2+^-free solution, while the recording electrode was filled with a K^+^-containing solution. To measure *I*_K(M)_ amplitude, we voltage-clamped each tested cell at −50 mV, and the depolarizing pulse up to 1 s in duration to −10 mV was imposed on it. The *I*_K(M)_ amplitude measured at the end of depolarizing pulses in the presence of 300 μM corylin was defined as 1.0 (i.e., 100%). The corresponding amplitudes obtained during the control period (in the absence of corylin) and during exposure to different concentrations of corylin (1–300 μM) were measured and compared. The concentration required to stimulate 50% of the current amplitude was determined according to a modified Hill function. That is,

(1) percentage increase=Emax×[C]nHEC50nH+[C]nH

In this equation, *EC_50_* = the concentration required for 50% stimulation; *n_H_* = the Hill coefficient; [*C*] = the corylin concentration applied; and *E*_max_ = maximal stimulation. This formula enables optimal convergence, providing the best fit line and accurate parameter estimates (e.g., *EC*_50_ and *n_H_*).

The activation time constants (τ_act_) of *I*_K(M)_ in response to prolonged membrane depolarization, obtained in the absence or presence of corylin, were determined by fitting the digitized current traces with a single-exponential function, as indicated in Figure 1B.

To characterize the stimulatory action of corylin on *I*_K(M)_ amplitude, we constructed the quasi-steady-state activation curve of the current. The relationships between the membrane potentials and the normalized amplitudes of *I*_K(M)_ with or without the existence of this compound were established and thereafter fitted with a Boltzmann function given by:

IImax=11+e−(V−V1/2)qF(RT)

where *I*_max_ = the maximal activated current of *I*_K(M)_; *V* = the membrane potential; *V*_1/2_ = the voltage for half-maximal stimulation; *q* = the apparent gating charge of the activation curve of *I*_K(M)_; *F* = Faraday’s constant; *R* = the universal gas constant; and *T* = the absolute temperature.

Linear (e.g., single-channel conductance) and nonlinear (e.g., concentration-dependent relationships and voltage-dependent activation curves) fittings were performed on the datasets using an interactive least-squares approach. Data analysis was carried out with software tools including the Solver add-in in Excel^®^ 2021 (Microsoft) and OriginPro^®^ 2026 (OriginLab).

### 4.5. Single-Channel Analysis of the K_M_ Channel

Single K_M_-channel currents in GH_3_ cells were recorded and analyzed by pClamp 10.7 (Molecular Devices). To evaluate single-channel opening events, amplitude distributions were fitted with multi-Gaussian adjustments. Channel open probabilities were determined through an iterative process to minimize the χ^2^ values across a sufficiently large set of independent observations. Open lifetime distributions of K_M_ channels (i.e., mean open time), obtained with or without corylin’s presence, were fitted using least-squares analysis with logarithmically scaled bin widths.

### 4.6. Statistical Analyses

All data are presented as the mean ± standard error of the mean (SEM), where n denotes the number of cells sampled. The normality of data distribution was evaluated using appropriate statistical tests. Differences between the two groups were analyzed using either paired or unpaired Student’s *t*-tests, depending on the experimental design. For comparisons involving multiple groups, one-way or two-way analysis of variance (ANOVA) was applied, with repeated-measures designs used when appropriate. When significant effects were detected, Fisher’s least-significant difference (LSD) post hoc test was performed for pairwise comparisons. A probability of *p* < 0.05 was considered statistically significant, and significance levels are denoted in the figure by *, **, or †.

## 5. Conclusions

This study demonstrates that corylin, a bioactive flavonoid, enhances *I*_K(M)_ in GH_3_ cells in a manner dependent on concentration, membrane voltage, and Hys_(V)_. In the presence of corylin, the activation curve of *I*_K(M)_ is shifted leftward, indicating facilitated channel activation. Additionally, corylin increases the open-state probability of K_M_ channels, accompanied by a prolongation of the mean open time. Docking analysis suggests that the corylin-induced activation of K_M_ channels is likely associated with its binding to KCNQ2 channels. Together, these effects may influence the regulation of electrical activity in various excitable cells under physiological conditions in vivo.

## Data Availability

The original contributions presented in this study are included in the article. Further inquiries can be directed to the corresponding author(s).

## Abbreviations

The following abbreviations are used in this manuscript:

ANOVA	Analysis of variance
AP	Action potential
Carv	Carvedilol
Corylin	3-(2,2-dimethylchromen-6-yl)-7-hydroxychromen-4-one
Dapa	Dapagliflozin
EC_50_	Concentration required for 50% stimulation
*Erg*	*Ether*-*à*-*go*-*go*-related gene
HERG	Human *ether*-*à*-*go*-*go*-related gene
Hys_(V)_	Voltage-dependent hysteresis;
*I*-*V*	Current versus voltage
*I*_K(erg)_	*Erg*-mediated K^+^ current
*I*_K(M)_	M-type K^+^ current
K_M_ channel	M-type K^+^ (KCNQ/K_7_) channel
Lino	Linopirdine
LSD test	Least-significant difference test
SEM	Standard error of mean
TTX	Tetrodotoxin
τ_act_	Activation time constant
V_ramp_	Ramp voltage
